# Prediction of pneumonia hospitalization in adults using health checkup data

**DOI:** 10.1371/journal.pone.0180159

**Published:** 2017-06-29

**Authors:** Hironori Uematsu, Kazuto Yamashita, Susumu Kunisawa, Tetsuya Otsubo, Yuichi Imanaka

**Affiliations:** Department of Healthcare Economics and Quality Management, Graduate School of Medicine, Kyoto University, Yoshida Konoe-cho, Sakyo-ku, Kyoto City, Kyoto, Japan; Azienda Ospedaliero Universitaria Careggi, ITALY

## Abstract

**Objectives:**

Community-acquired pneumonia is a common cause of hospitalization, and pneumococcal vaccinations are recommended for high-risk individuals. Although risk factors for pneumonia have been identified, there are currently no pneumonia hospitalization prediction models based on the risk profiles of healthy subjects. This study aimed to develop a predictive model for pneumonia hospitalization in adults to accurately identify high-risk individuals to facilitate the efficient prevention of pneumonia.

**Methods:**

We conducted a retrospective database analysis using health checkup data and health insurance claims data for residents of Kyoto prefecture, Japan, between April 2010 and March 2015. We chose adults who had undergone health checkups in the first year of the study period, and tracked pneumonia hospitalizations over the next 5 years. Subjects were randomly divided into training and test sets. The outcome measure was pneumonia hospitalization, and candidate predictors were obtained from the health checkup data. The prediction model was developed and internally validated using a LASSO logistic regression analysis. Lastly, we compared the new model with comparative models.

**Results:**

The study sample comprised 54,907 people who had undergone health checkups. Among these, 921 were hospitalized for pneumonia during the study period. The c-statistic for the prediction model in the test set was 0.71 (95% confidence interval: 0.69–0.73). In contrast, a comparative model with only age and comorbidities as predictors had a lower c-statistic of 0.55 (95% confidence interval: 0.54–0.56).

**Conclusions:**

Our predictive model for pneumonia hospitalization performed better than comparative models, and may be useful for supporting the development of pneumonia prevention measures.

## Introduction

Community-acquired pneumonia is a major cause of hospitalization and death in aging societies [1,2]. Japan is currently the world’s foremost super-aging society, with elderly people aged 65 years and older accounting for 26.7% of the population in 2015 [3]. Due to the increased susceptibility of elderly people to pneumonia, it is crucial for policymakers in Japan and other aging societies to develop appropriate and efficient strategies to prevent and treat this disease.

The Infectious Disease Society of America (IDSA) and the American Thoracic Society (ATS) recommend the use of pneumococcal vaccines as a major preventive measure because *Streptococcus pneumoniae* is thought to be the predominant pathogen in community-acquired pneumonia [4,5]. According to IDSA/ATS guidelines, pneumococcal vaccines are recommended for adults at high risk of pneumococcal diseases, such as persons aged over 65 years or those with specific existing conditions [4].

Susceptibility to pneumonia or increased disease severity in elderly persons is dependent on their individual risk profiles, such as underlying medical conditions and lifestyle [6,7]. Thus, healthy elderly persons who have no risk factors other than age may be less susceptible to pneumonia than the average elderly person. In Japan, it has been reported that elderly persons in recent years are healthier when compared with those from earlier generations [8]. As a result, not all elderly people may require vaccinations against pneumonia. At the same time, there is a need to identify younger adults who may require vaccinations based on various risk factors [6].

The efficacy of pneumococcal vaccines, recommended by the current clinical guidelines, among the elderly and other high-risk adults is still unclear. A meta-analysis performed by Huss et al. showed that pneumococcal vaccination among the target population was not effective in preventing pneumonia [9]. Diao et al. also reported a similar result; however, it was found that the vaccination had weak effects in the high-risk people [10]. In addition, inappropriate use of the pneumococcal vaccine may unnecessarily expose people to possible adverse effects such as anaphylaxis [11]. From the broader point of view of benefits and safety, it would be more reasonable to focus on vaccinating people who actually have a high risk for pneumonia.

To identify people who would benefit from vaccination, prediction models are needed to assess the probability of an individual having pneumonia in the future. Although previous studies have identified pneumonia risk factors or developed prediction models for patients in clinical settings [6,12], there has yet to be any model for predicting the occurrence of pneumonia based on the risk profile of healthy subjects. Prediction models should monitor for pneumonia occurrence for approximately 5 years at least to determine whether an individual requires vaccination because revaccinations are considered in 5-year intervals [13].

In this study, we aimed to develop a predictive model for pneumonia hospitalization in adults using health checkup data in order to accurately identify high-risk individuals to facilitate the efficient prevention of pneumonia.

## Materials and methods

### Study design and data source

We conducted a retrospective analysis using a database comprising health checkup data and health insurance claims data for residents of Kyoto prefecture, Japan, between April 2010 and March 2015. In 2008, Japan’s Ministry of Health, Labour and Welfare initiated a nationwide “specific health checkup” program for the screening and management of lifestyle-related diseases. This program involves physical examinations and guidance sessions for adult insurance enrollees aged 40–74 years [14]. Eligible subjects have specific health check-ups done optionally once a year. Data from these health check-ups were used in our analysis because many relevant health check items related to pneumonia events were included in this data set.

The claims data comprised information that is periodically submitted from healthcare providers to National Health Insurance and Long-Life Medical Care Insurance, which are the 2 major insurance payers in Japan. National Health Insurance provides insurance coverage to persons who are self-employed, part-time workers, unemployed, and retirees. Long-Life Medical Care Insurance provides insurance coverage to elderly persons aged 75 years and older as well as disabled persons aged 65 to 74 years. The total number of insured persons enrolled in these 2 insurance systems accounted for over 40% of the Japanese population in 2013 [15].

Health checkup data include examinee identification number, date of health checkup, age, sex, body mass index (BMI), systolic and diastolic blood pressure (BP) measurements, abdominal girth, lifestyle, medications, comorbidities, symptoms, procedures such as funduscopic examination and electrocardiogram (ECG), blood tests, and urine tests. Claims data include patient identification number, dates of consultation, diagnosed disease during consultation, date of hospital admission, diagnosed disease on admission, and all medical care provided during hospitalization according to insurance medical fee payments. Diagnosed diseases were identified using International Classification of Diseases, 10th Revision (ICD-10) codes. The 2 data sources were merged into a single database using the subjects’ identification numbers.

### Subject inclusion and exclusion criteria

We selected people aged 40 to 74 years who had undergone specific health checkups between April 1, 2010 and March 31, 2011. If a subject had undergone multiple checkups within that year, we used data from the first checkup. We excluded cases who had the same identification number but different birth dates or sex.

### Outcome and predictors

In this study setting, the primary outcome was hospitalization for community-acquired pneumonia. We identified cases with records of major diagnoses (including suspected diagnoses) of pneumonia (corresponding ICD-10 codes: J10.0, J11.0, J12–J18, J69, A48.1, B01.2, B05.2, B37.1, and B59) on admission between April 1, 2010 and March 31, 2015. Candidate predictors were obtained from the specific health checkup data between April 1, 2010 and March 31, 2011. The follow-up period for each individual was 4–5 years, depending on the date of his/her initial health checkup. Missing values (if any) for each predictive variable were converted and categorized as dummy variables. Details of the candidate predictors and their respective cut-off points are summarized in Table 1. The cut-off points were determined according to the health checkup guidelines provided by the Ministry of Health, Labour and Welfare [16].

**Table 1:** Candidate predictors from specific health checkup data.

	Variable (unit)	Cut-off/Annotation
Demographic	Age (years)	40–49 (ref), 50–59, 60–69, 70–74
	Sex	Male, Female (ref)
Physical	BMI (kg/m^2^)	<18.5, 18.5–25 (ref), ≥25
	Systolic BP (mmHg)	<130 (ref), 130–140, ≥140
	Diastolic BP (mmHg)	<85 (ref), 85–90, ≥90
	Abdominal girth (m)	Male
		<0.85 (ref), ≥0.85
		Female
		<0.90 (ref), ≥0.90
Lifestyle	Body weight increase	≥10 kg increase from weight at age 20
		Yes, No (ref)
	Daily exercise exceeding 30 mins	Exercise that induces light perspiration for at least 30 minutes per session, twice weekly for over a year
		Yes, No (ref)
	Daily walking	Walking (or an equivalent amount of physical activity) for more than one hour a day
		Yes, No (ref)
	Walking Speed	Faster than other people of the same age and sex as the subject
		Yes, No (ref)
	Body weight change	Change of ±3 kg in a year
		Yes, No (ref)
	Eating speed	Eating speed relative to other people of the same sex as the subject
		Fast, Normal (ref), Slow
	Eating before sleep	Eating within 2 hours before sleeping over 3 times a week
		Yes, No (ref)
	Midnight meals	Eating again after dinner over 3 times a week
		Yes, No (ref)
	Not having breakfast	Not having breakfast over 3 times a week
		Yes, No (ref)
	Current smoker	Smoked over 100 cigarettes or have smoked over 6 months, and have been smoking over a month
		Yes, No (ref)
	Frequency of alcohol consumption	Daily, Sometimes, Rare (ref)
	Amount of alcohol consumption (L/day)	Amount of alcoholic drinks (15% alcohol by volume) consumed per day
		<0.18 (ref), 0.18–0.36, 0.36–0.54, ≥0.54
	Sleep Duration	Adequate, Inadequate (ref)
Medication	Antihypertensive drug	Prescribed, Not prescribed (ref)
	Hypoglycemic drug	Prescribed, Not prescribed (ref)
	Antidyslipidemic drug	Prescribed, Not prescribed (ref)
Comorbidities	Comorbidity (Any)	Yes, No (ref)
	Cerebrovascular disease	Yes, No (ref)
	Heart disease	Yes, No (ref)
	Renal failure	Yes, No (ref)
Symptoms	Subjective symptoms	Yes, No (ref)
	Objective symptoms	Yes, No (ref)
Procedures	Funduscopy ^a^	Any abnormalities,
		No abnormalities (ref)
	ECG ^a^	Any abnormalities,
		No abnormalities (ref)
Blood Test	TG (mmol/L)	<1.70 (ref), 1.70–3.39, ≥3.39
	HDL (mmol/L)	≤0.88, 0.88–1.01, >1.01 (ref)
	LDL (mmol/L)	<3.10 (ref), 3.10–3.62, ≥3.62
	GOT (U/L)	<31 (ref), 31–51, ≥51
	GPT (U/L)	<31 (ref), 31–51, ≥51
	γ-GTP (U/L)	<51 (ref), 51–101, ≥101
	Fasting blood glucose (mmol/L)	<55.10(ref), 55.10–69.43, ≥69.43
	HbA1c (mmol/mol)	<37.71 (ref), 37.71–47.55, ≥47.55
	Hb ^a^ (g/L)	Male
		≤120, 120–130, >130 (ref)
		Female
		≤110, 110–120, >120 (ref)
Urine Test	Glycosuria (mmol/L)	Positive (≥2.78), Negative (ref)
	Proteinuria (g/L)	Positive (≥0.15), Negative (ref)

BMI: Body Mass Index, BP: Blood Pressure, ECG: Electrocardiogram, TG: Triglyceride, HDL: High-density Lipoprotein Cholesterol, LDL: Light-density Lipoprotein Cholesterol, GOT: Glutamic Oxaloacetic Transaminase, GPT: Glutamate Pyruvic Transaminase, γ-GTP: γ-glutamyl Transpeptidase, HbA1c: Hemoglobin A1c, Hb: Hemoglobin

^a^ Funduscopy, ECG, and Hb tests were only performed when ordered by physicians.

### Statistical analysis

First, we randomly divided our study sample equally into a training dataset and a test dataset. We adopted the Least Absolute Shrinkage and Selection Operator (LASSO) logistic analytical approach, which performs both variable selection and regularization in order to enhance prediction accuracy and interpretability of the model [17]. The variable selection reduces multicollinearity in cases where correlations among variables are strong [18]. We performed 10-fold cross validation for selecting the LASSO tuning parameter using the training dataset [17], and developed the model based on the odds ratios of the predictors. Next, we applied the fitted model to the test dataset for internal validation, and calculated the c-statistic and the calibration slope. The slope was estimated by plotting the observed frequency by the decile of the predicted probabilities [19]. In addition, we calculated sensitivity, specificity, positive predictive value (PPV) and negative predictive value (NPV) of the model for the optimal threshold at the point closest to the top-left part of the receiver operating characteristic curve that indicates perfect sensitivity or specificity. Finally, we compared this LASSO logistic prediction model to 2 comparative models based on published guidelines for the pneumococcal vaccine: the first model used age (65 years and older) as the predictor, and the second model used age (65 years and older) and comorbidities as predictors [4].

All statistical analyses were performed using R statistical software (version 3.3.0) and the R package “glmnet” (version 2.0–5) was used for implementing the LASSO analysis [20]. The Ethics Committee of Kyoto University Graduate School of Medicine approved the collection and analysis of the specific health checkup data and health insurance claims data (Approval Number: E-1023). In accordance with the Japanese Ethical Guidelines for Epidemiological Research, our study waived the need for informed consent.

## Results

We identified a total of 55,842 candidate subjects using the inclusion criteria. After excluding 934 cases with contradictory information for birth dates and sex, there remained 54,908 people. Another case had a missing value in the “objective symptoms” in the data, and its exclusion resulted in a total of 54,907 subjects used in analysis. The mean and median ages of the subjects were 64.6 years and 67.0 years, respectively. There were 22,830 female (41.6%) in the sample. There were 921 hospitalizations (1.7%) among the subjects during the 5-year study period.

### Predictors

Table 2 shows the odds ratios for the various predictors of pneumonia hospitalization calculated using the LASSO logistic regression analysis in the training sample (n = 27,454). Variables found to be positively associated with pneumonia hospitalization were older age (70–74 years and 60–69 years), male gender, current smoking status, low hemoglobin count, cerebrovascular disease, low BMI (<18.5 kg/m^2^), abnormal ECG findings (any findings), presence of comorbidities (any comorbidities), and body weight change (the other positive predictors are shown in Table 2). Variables that were negatively associated with pneumonia hospitalization were walking speed (fast), aged 50–59 years, daily exercise exceeding 30 minutes, high BMI (>25kg/m^2^), high LDL (3.10–3.62 mmol/L), daily alcohol consumption (0.18–0.36 L), alcohol consumption frequency (occasional), eating speed (fast), high systolic BP (130–140 mmHg), daily walking, GOT (31–51 U/L) and body weight increase (missing data). Parameter estimates of several explanatory variables (such as diastolic BP and sleep duration) were shrunk to zero during the LASSO variable selection indicating little or no impact on predicting pneumonia hospitalization (data not shown).

**Table 2:** Predictors of pneumonia hospitalization.

Odds Ratios in descending order		Odds Ratios in ascending order	
Variables	Odds Ratio ^a^	Variables	Odds Ratio ^a^
Age 70–74 y	1.390	Walking Speed. fast	0.857
Sex, male	1.361	Age 50–59 y	0.926
Age 60–69 y	1.222	Daily exercise exceeding 30 minutes	0.937
Current Smoker	1.163	BMI >25kg/m^2^	0.947
Hb ≤120 g/L (Male) or Hb ≤110 g/L (Female)	1.146	LDL 3.10–3.62 mmol/L	0.948
Cerebrovascular disease	1.125	Amount of alcohol consumption 0.18–0.36 L/day	0.955
BMI <18.5kg/m^2^	1.099	Frequency of alcohol consumption, sometimes	0.967
ECG, Any abnormalities	1.082	Systolic BP 130–140 mmHg	0.970
Comorbidities (Any)	1.075	Eating speed, fast	0.975
Body weight change	1.074	Daily walking	0.990
γ-GTP ≥101 U/L	1.074	GOT 31–51 U/L	0.993
Funduscopy, missing data	1.070	Body weight increase data, missing data	0.999
GOT ≥ 51U/L	1.068		
HbA1c 37.71–47.55 mmol/mol	1.058		
Hb 120–130 g/L (Male) or Hb 110–120 g/L (Female)	1.053		
Glycosuria	1.052		
Proteinuria	1.051		
Objective symptoms	1.050		
Fasting blood glucose ≥ 69.43mmol/L	1.045		
Hypoglycemic drugs	1.039		
Glycosuria, missing data	1.039		
HDL ≤0.88 mmol/L	1.035		
Eating speed, slow	1.025		
HDL 0.88–1.01 mmol/L	1.023		
HbA1c ≥47.55 mmol/mol	1.022		
Renal failure	1.011		
Heart disease	1.007		
Proteinuria, missing data	1.001		

^a^ Confidence intervals are not shown because the odds ratios were calculated using LASSO logistic regression analysis.

### Model evaluation

**Fig 1:**
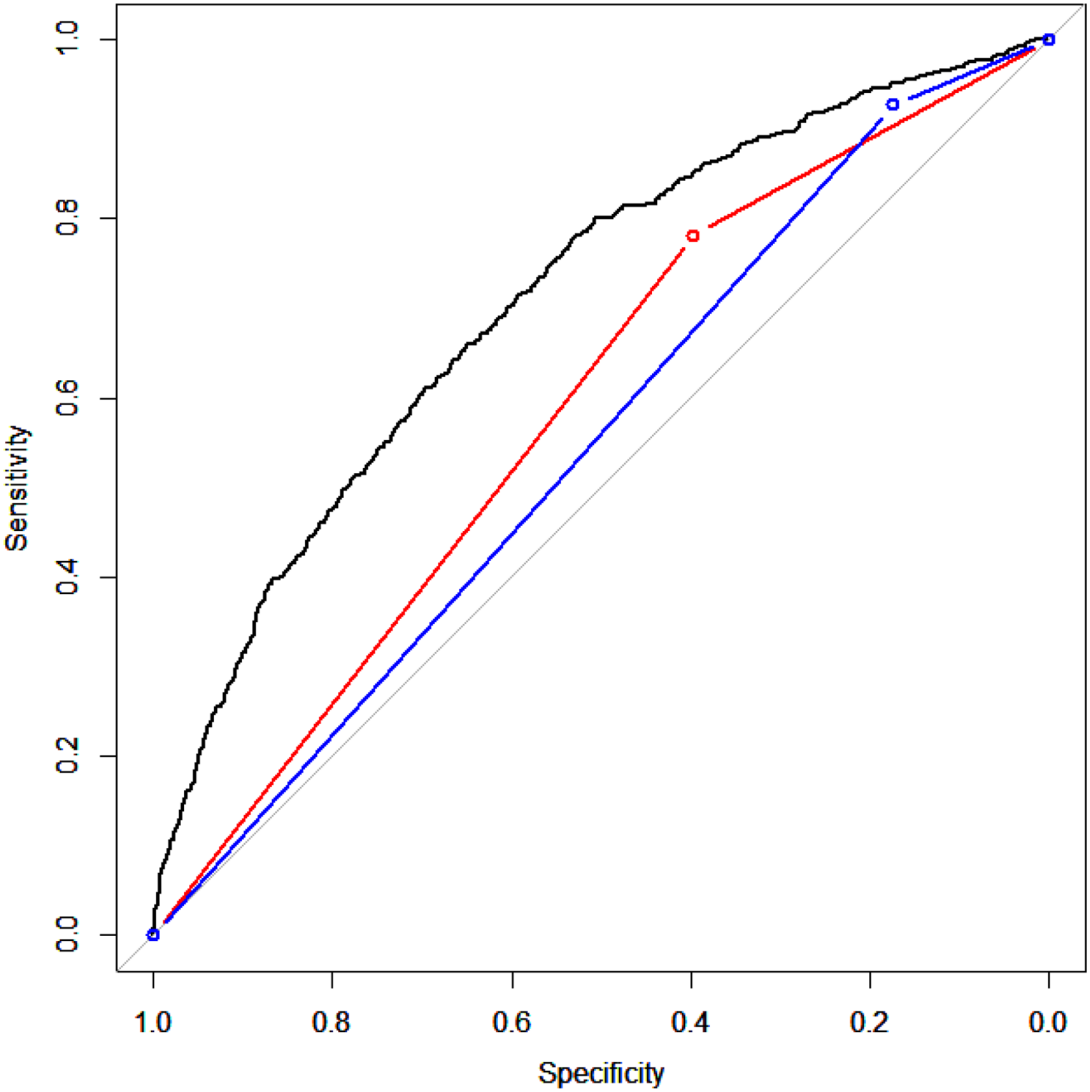
Receiver operating characteristic curves of the new and existing pneumonia hospitalization prediction models. The distortion curve indicates all cut-off points of our new model. The red line indicates Comparative Model 1, and the blue line indicates Comparative Model 2.

**Fig 2:**
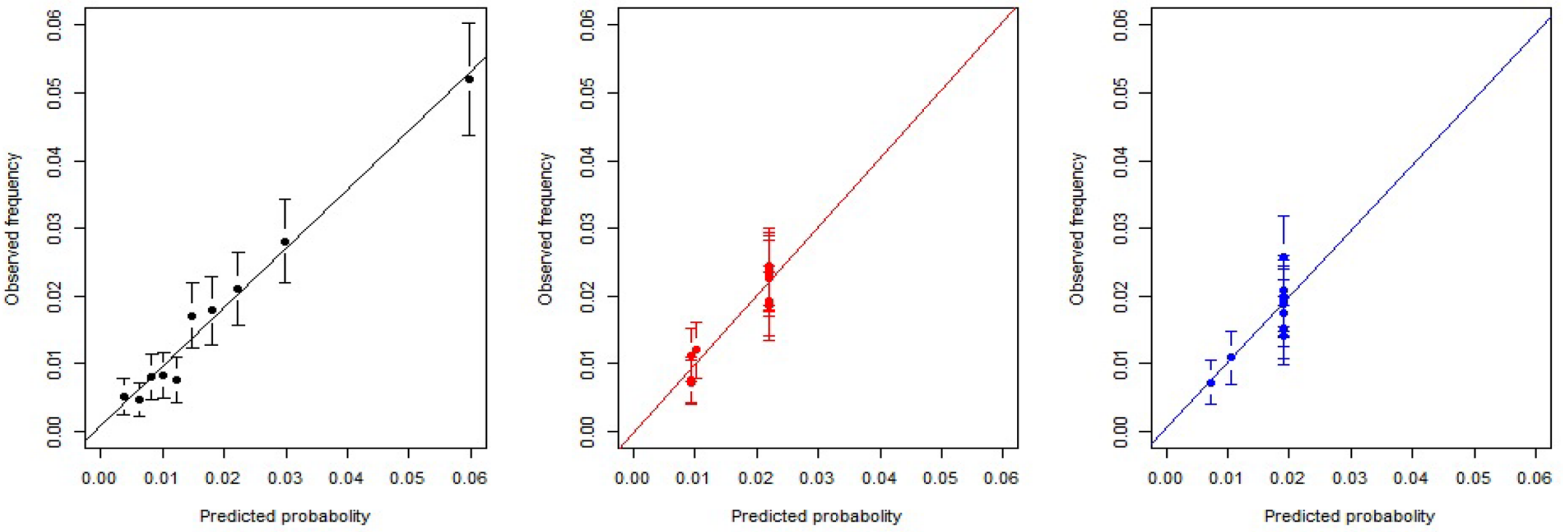
Calibration plots and slopes of the new and existing pneumonia hospitalization prediction models. The dots represent the observed frequency of pneumonia hospitalization in 10 groups divided by similar predicted probabilities, and a straight line through the dots represents the calibration slope. The bars represent 95% confidence intervals of the proportion of the observed frequency of pneumonia hospitalization in the 10 groups. The black dots and bars indicate our new model, the red indicate Comparative Model 1, and the blue indicate Comparative Model 2.

**Fig 3:**
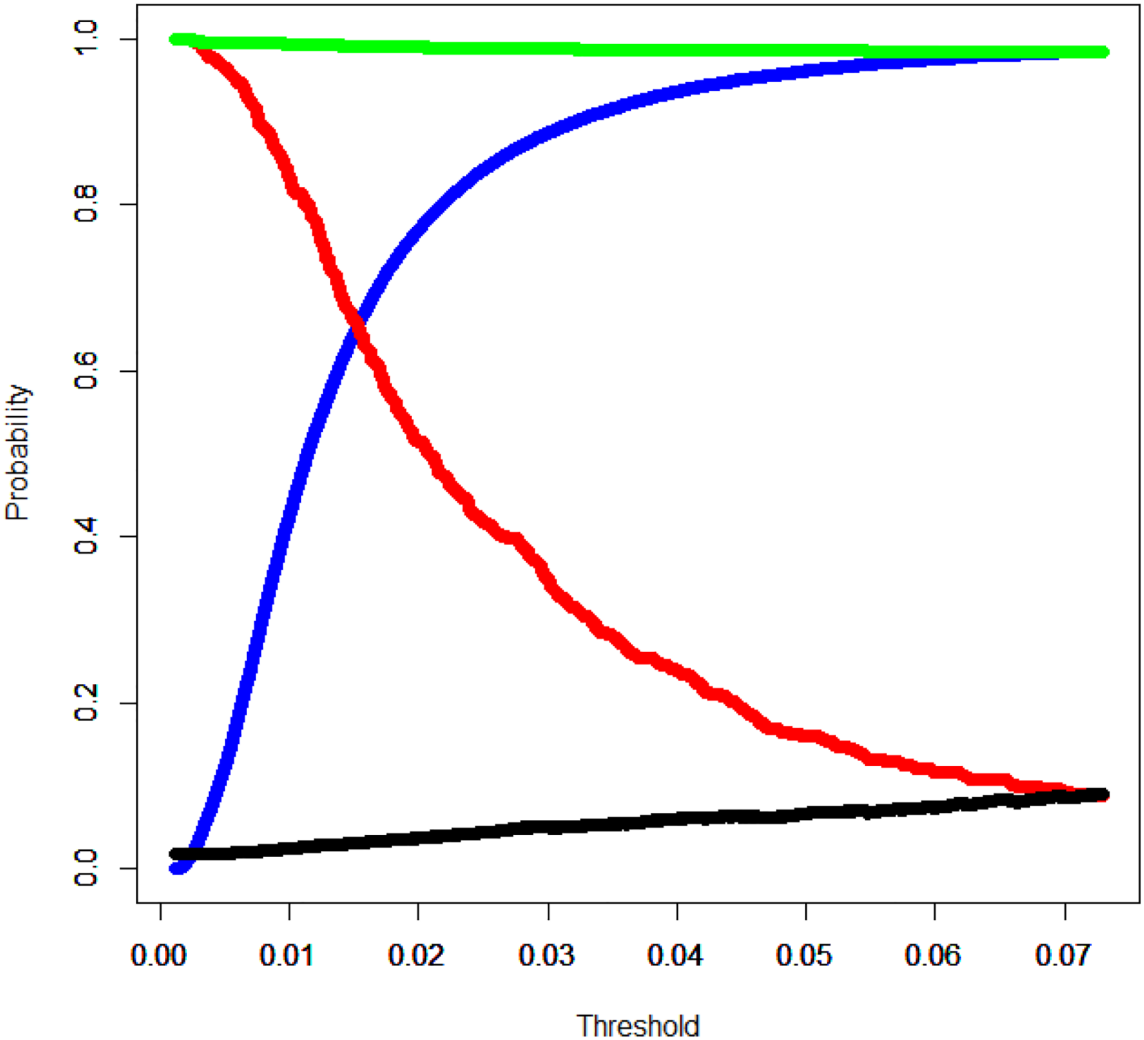
Model performance at various thresholds. The figure shows the sensitivity, specificity, negative predictive values, and positive predictive values of our new model at various thresholds. The red line indicates sensitivity, the blue line indicates specificity, the green line indicates the negative predictive value, and the black line indicates the positive predictive value.

Table 3 shows a comparison of model evaluations in the test sample (n = 27,453). The c-statistic of our newly developed model was 0.71 (95% confidence interval: 0.69–0.73) and the calibration slope was 0.88 (95% confidence interval, 0.79–0.95). At the optimal threshold of 0.015, we calculated the sensitivity, specificity, PPV, and NPV to be 0.660, 0.650, 0.032, and 0.991, respectively. When we set the sensitivity of our model to be equal to those of the comparative models based on current guidelines, we found that our model had superior (albeit not significantly) specificity, PPV, and NPV relative to the comparative models. Fig 1 presents the receiver operating characteristic curves of the 3 models, which demonstrate that the comparative models had lower predictive performance at all cut-off points than our model. Fig 2 presents the calibration dots and the slopes of the 3 models, and it shows that the comparative models estimated the slopes less accurately than our model. Fig 3 shows the changes in sensitivity, specificity, PPV, and NPV for different thresholds for predicting pneumonia. When the threshold increased, sensitivity decreased, specificity increased, NPV gradually decreased, and PPV gradually increased. These findings indicate that lowering the threshold of the model reduces the risk of overlooking pneumonia cases.

**Table 3:** Comparison of model evaluations in the test settings.

	Sensitivity	Specificity	PPV	NPV	C-statistic	Calibration slope
New model (Optimal threshold)	0.660	0.650	0.032	0.991	0.71 (95%CI: 0.69–0.73)	0.88 (95%CI: 0.79–0.95)
Comparative Model 1	0.782	0.397	0.022	0.991	0.60 (95%CI: 0.58–0.62)	1.01 (95%CI: 0.77–1.25)
Comparative Model 2	0.927	0.174	0.019	0.993	0.55 (95%CI: 0.54–0.56)	0.97 (95%CI: 0.47–1.47)
New model (Sensitivity = 0.782)	0.782	0.522	0.028	0.993	0.71 (95%CI: 0.69–0.73)	0.88 (95%CI: 0.79–0.95)
New model (Sensitivity = 0.927)	0.927	0.233	0.021	0.995	0.71 (95%CI: 0.69–0.73)	0.88 (95%CI: 0.79–0.95)

PPV: Positive Predictive Value, NPV: Negative Predictive Value, CI: Confidence Intervals

New model: Our model developed using specific health checkup data

Comparative Model 1: Predictor used was age (65 y and older) only

Comparative Model 2: Predictors used were age (65 y and older) and comorbidities only

## Discussion

In an analysis of 54,907 adults using health checkup data and health insurance claims data, we found that hospitalization for community-acquired pneumonia occurred in 1.68% of the subjects over a 5-year period. We developed and internally validated a predictive model for pneumonia hospitalization, which demonstrated a moderate level of predictive power (c-statistic: 0.71; 95% confidence intervals: 0.69–0.73). In addition to age and comorbidities, we identified several other predictors of pneumonia hospitalization from information available in the specific health checkup data. These findings indicate a need for personalized and comprehensive evaluations to identify adults at high risk of pneumonia more accurately than comparative evaluations.

To the best of our knowledge, this is the first study to develop a model for predicting pneumonia hospitalization in healthy subjects using routine health checkup data. Previous studies have identified several risk factors of community-acquired pneumonia that support our findings of the associations between various factors and pneumonia hospitalization [6]. For example, age, the male sex, comorbidities, alcohol consumption, smoking, and low BMI were identified as potent predictors of pneumonia in both our model as well as in previous studies [6]. Our analysis also found ECG abnormalities, high gamma-glutamyl transpeptidase levels, high glutamate oxaloacetic transaminase levels, high hemoglobin A1c levels, glycosuria, proteinuria, and high fasting blood glucose levels to be potent predictors of pneumonia. These variables may be indicative of specific underlying conditions in subjects such as heart disease, liver disease, kidney disease, and diabetes mellitus. Previous studies have also reported associations between these comorbidities and pneumonia [6]. The majority of predictors included in our model were therefore supported by current literature.

On the other hand, some predictors in our model had less reinforcement from existing evidence. For example, anemia had a relatively strong association with pneumonia in our model, but few studies have reported this association. Almario et al. found that pernicious anemia was a risk factor for community-acquired pneumonia [21]. Low serum high-density lipoprotein (HDL) levels can lead to arteriosclerosis, and our model discovered that it slightly increased the risk of pneumonia hospitalization. Chien et al. have reported that low HDL levels are a predictor of mortality in patients with severe community-acquired pneumonia [22]. These previous studies therefore indicate that anemia and low serum HDL levels have the potential to be predictors of pneumonia hospitalization, but have provided little direct evidence. Further studies are required to confirm the effects of these factors.

Although our model only had moderate predictive power, this may be further improved by using other potent predictors that were not available in this study. For example, previous studies have identified the following to be important risk factors of pneumonia: poor dental hygiene, communal living, previous respiratory infection, drug use (benzodiazepines, steroids, and opioids), contact with children, and low socioeconomic status [6,23–26]. In addition, more detailed information on comorbidities may also improve the model’s predictive performance. The inclusion of these critical variables to standard health checkup data would improve our prediction model by strengthening its predictive power and accuracy.

Due to the inclusion of various other factors, our prediction model had better discrimination than existing guideline-based models that only included age (65 years and older) or age and comorbidities [4]. Our analysis found that healthy elderly people aged 65 years and older had a low susceptibility to pneumonia, indicating that there is little need to administer pneumococcal vaccinations to these individuals. Accordingly, public health agencies and healthcare providers may be able to conduct vaccinations on a case-by-case basis using personal health data.

Because there is a large variety of possible predictors available from health data, our prediction model allows for various thresholds to be set for distinguishing pneumonia events according to desired levels of sensitivity or specificity. As Fig 3 shows, we can increase PPV and specificity if the threshold is increased or sensitivity is decreased. Required sensitivity or specificity levels may vary among countries or groups of subjects. Analysts can therefore stipulate their desired performance when using this prediction model to enable better decision making relative to existing methods that incorporate only subject age or comorbidities. We therefore believe that our new model helps to identify subjects with a greater need for the vaccines.

Our study has several limitations. First, the specific health checkup data did not include information about any previous inoculations with the pneumococcal vaccine and its antibody response. As these vaccinations can contribute to reducing the occurrence of pneumonia in high-risk groups [27], the lack of this information may introduce bias into the estimates of the predictors. However, the percentage of pneumococcal vaccinations was 7.7% in elderly people in Japan in 2009, which was much lower than other developed countries [28]. This may therefore reduce the possible bias. Second, the study may be susceptible to selection bias as we did not use population-based data, and participation in the specific health checkups is optional. Future application of this model to health checkup data from a wider population would help to alleviate the effects of this bias. Third, our study may underestimate the prevalence of pneumonia because the health insurance claims data are unable to track subjects who die, move out of Kyoto prefecture, or shift to another health insurance plan. Fourth, due to data limitation, we did not validate our model externally, to confirm whether the model performance could be optimistic. It is therefore necessary to validate the model externally by using data from different periods or geographical areas in order to ensure validation of the model in future research.[29] Fifth, it is difficult to interpret all explanatory variables used in the analysis, because of the variable selection of the LASSO analysis. Finally, the subjects may not be representative of all hospitalized cases with community-acquired pneumonia because we did not include those whose major diagnosis was either sepsis or respiratory failure with a secondary diagnosis of pneumonia. This may result in a possible underestimation of pneumonia cases.

## Conclusions

This study developed and internally validated a model to predict pneumonia hospitalization based on specific health checkup data. We had hypothesized that the inclusion of detailed risk profiles in addition to age and comorbidities would enable more accurate predictions of future pneumonia events. Our findings support this hypothesis, and future improvements to the model may facilitate efficient prevention of pneumonia in an aging society.

## References

[1] Brown JS. Community-acquired pneumonia. Clin.Med.(Lond) 2012;12:538–543. 23342408 10.7861/clinmedicine.12-6-538 PMC5922594

[2] Remington LT, Sligl WI. Community-acquired pneumonia. Curr.Opin.Pulm.Med. 2014;20:215–224. doi: 10.1097/MCP.0000000000000052 24614242 10.1097/MCP.0000000000000052

[3] The Statistics Bureau of Japan. URL http://www.stat.go.jp/data/topics/topi901.htm (accessed 2017-03-03) (in Japanese).

[4] Mandell LA, Wunderink RG, Anzueto A, Bartlett JG, Campbell GD, Dean NC, et al Infectious Diseases Society of America/American Thoracic Society consensus guidelines on the management of community-acquired pneumonia in adults. Clin.Infect.Dis. 2007;44 Suppl 2:S27–72. 17278083 10.1086/511159 PMC7107997

[5] van der Poll T, Opal SM. Pathogenesis, treatment, and prevention of pneumococcal pneumonia. The Lancet 2009;374:1543–1556. 10.1016/S0140-6736(09)61114-4 19880020

[6] Torres A, Peetermans WE, Viegi G, Blasi F. Risk factors for community-acquired pneumonia in adults in Europe: a literature review. Thorax 2013;68:1057–1065. doi: 10.1136/thoraxjnl-2013-204282 24130229 10.1136/thoraxjnl-2013-204282 PMC3812874

[7] Uematsu H, Kunisawa S, Sasaki N, Ikai H, Imanaka Y. Development of a risk-adjusted in-hospital mortality prediction model for community-acquired pneumonia: a retrospective analysis using a Japanese administrative database. BMC Pulmonary Medicine 2014;14:203 doi: 10.1186/1471-2466-14-203 25514976 10.1186/1471-2466-14-203 PMC4279890

[8] Orimo H, Ito H, Suzuki T, Araki A, Hosoi T, Sawabe M. Reviewing the definition of “elderly”. Geriatrics & gerontology international 2006;6:149–158.

[9] Huss A, Scott P, Stuck AE, Trotter C, Egger M. Efficacy of pneumococcal vaccination in adults: a meta-analysis. CMAJ 2009;180:48–58. doi: 10.1503/cmaj.080734 19124790 10.1503/cmaj.080734 PMC2612051

[10] Diao W, Shen N, Yu P, Liu B, He B. Efficacy of 23-valent pneumococcal polysaccharide vaccine in preventing community-acquired pneumonia among immunocompetent adults: A systematic review and meta-analysis of randomized trials. Vaccine 2016;34:1496–1503. doi: 10.1016/j.vaccine.2016.02.023 26899376 10.1016/j.vaccine.2016.02.023

[11] Miller ER, Moro PL, Cano M, Lewis P, Bryant-Genevier M, Shimabukuro TT. Post-licensure safety surveillance of 23-valent pneumococcal polysaccharide vaccine in the Vaccine Adverse Event Reporting System (VAERS), 1990–2013. Vaccine 2016;34:2841–2846. doi: 10.1016/j.vaccine.2016.04.021 27087150 10.1016/j.vaccine.2016.04.021 PMC6546117

[12] Schierenberg A, Minnaard MC, Hopstaken RM, van de Pol Alma C, Broekhuizen BD, de Wit NJ, et al External Validation of Prediction Models for Pneumonia in Primary Care Patients with Lower Respiratory Tract Infection: An Individual Patient Data Meta-Analysis. PloS one 2016;11:e0149895 doi: 10.1371/journal.pone.0149895 26918859 10.1371/journal.pone.0149895 PMC4769284

[13] Whiteside J, Grover M, Hitchcock K. Should patients receive 23-valent pneumococcal vaccination more than once? Clinical Inquiries, 2006 (MU) 2006. 16948967

[14] Ministry of Health, Labour, and Welfare, Japan Standard program of health checkup and health guidance (2007). URL http://www.mhlw.go.jp/bunya/shakaihosho/iryouseido01/info03a.html (accessed 2016-07-11) (in Japanese).

[15] Social Security Statistics Annual Database (2016). URL http://www.ipss.go.jp/ssj-db/ssj-db-top.asp (accessed 2016-07-07) (in Japanese).

[16] Ministry of Health, Labour, and Welfare, Japan Standard program of health checkup and health guidance, health chekups (2007). URL http://www.mhlw.go.jp/bunya/kenkou/seikatsu/pdf/02b.pdf (accessed 2016-07-11) (in Japanese).

[17] Tibshirani R. Regression shrinkage and selection via the lasso. Journal of the Royal Statistical Society.Series B (Methodological) 1996;:267–288.

[18] Chong I, Jun C. Performance of some variable selection methods when multicollinearity is present. Chemometrics Intellig.Lab.Syst. 2005;78:103–112.

[19] Steyerberg EW, Vickers AJ, Cook NR, Gerds T, Gonen M, Obuchowski N, et al Assessing the performance of prediction models: a framework for traditional and novel measures. Epidemiology 2010;21:128–138. doi: 10.1097/EDE.0b013e3181c30fb2 20010215 10.1097/EDE.0b013e3181c30fb2 PMC3575184

[20] Friedman J, Hastie T, Tibshirani R. glmnet: Lasso and elastic-net regularized generalized linear models. Version 2.0–5 [glmnet archive]. URL http://cran.r-project.org/web/packages/glmnet/index.html (accessed 2017-02-22).

[21] Almario CV, Metz DC, Haynes K, Yang YX. Risk of community-acquired pneumonia in patients with a diagnosis of pernicious anemia: a population-based retrospective cohort study. Eur.J.Gastroenterol.Hepatol. 2015;27:1259–1264. doi: 10.1097/MEG.0000000000000444 26225868 10.1097/MEG.0000000000000444 PMC4586398

[22] Chien Y, Chen C, Hsu C, Chen K, Yu C. Decreased serum level of lipoprotein cholesterol is a poor prognostic factor for patients with severe community-acquired pneumonia that required intensive care unit admission. J.Crit.Care 2015;30:506–510. doi: 10.1016/j.jcrc.2015.01.001 25702844 10.1016/j.jcrc.2015.01.001

[23] Almirall J, Bolibar I, Serra-Prat M, Roig J, Hospital I, Carandell E, et al New evidence of risk factors for community-acquired pneumonia: a population-based study. Eur.Respir.J. 2008;31:1274–1284. doi: 10.1183/09031936.00095807 18216057 10.1183/09031936.00095807

[24] Obiora E, Hubbard R, Sanders RD, Myles PR. The impact of benzodiazepines on occurrence of pneumonia and mortality from pneumonia: a nested case-control and survival analysis in a population-based cohort. Thorax 2013;68:163–170. doi: 10.1136/thoraxjnl-2012-202374 23220867 10.1136/thoraxjnl-2012-202374

[25] Dublin S, Walker RL, Jackson ML, Nelson JC, Weiss NS, Korff M, et al Use of Opioids or Benzodiazepines and Risk of Pneumonia in Older Adults: A Population-Based Case–Control Study. J.Am.Geriatr.Soc. 2011;59:1899–1907. doi: 10.1111/j.1532-5415.2011.03586.x 22091503 10.1111/j.1532-5415.2011.03586.x PMC3223721

[26] Giorgi Rossi P, Agabiti N, Faustini A, Ancona C, Tancioni V, Forastiere F, et al The burden of hospitalised pneumonia in Lazio, Italy, 1997–1999. The International Journal of Tuberculosis and Lung Disease 2004;8:528–536. 15137527

[27] Taneja C, Haque N, Oster G, Shorr AF, Zilber S, Kyan PO, et al Clinical and economic outcomes in patients with community-acquired Staphylococcus aureus pneumonia. Journal of Hospital Medicine 2010;5:528–534. doi: 10.1002/jhm.704 20734457 10.1002/jhm.704

[28] Ishida T. Efficacy of Pneumococcal Vaccine in Elderly (2011). URL https://www.jstage.jst.go.jp/article/jsrcr/21/3/21_245/_pdf (accessed 2016-07-12) (in Japanese).

[29] Debray TP, Vergouwe Y, Koffijberg H, Nieboer D, Steyerberg EW, Moons KG. A new framework to enhance the interpretation of external validation studies of clinical prediction models. J.Clin.Epidemiol. 2015;68:279–289. doi: 10.1016/j.jclinepi.2014.06.018 25179855 10.1016/j.jclinepi.2014.06.018

